# Unraveling BOLD-100 synergistic potential in pleural mesothelioma treatment: an in vitro study

**DOI:** 10.1007/s10637-025-01540-9

**Published:** 2025-05-08

**Authors:** Gregorio Bonsignore, Elia Ranzato, Simona Martinotti

**Affiliations:** 1 DiSIT- Dipartimento di Scienze e Innovazione Tecnologica, University of Piemonte Orientale, Viale Teresa Michel 11, 15121 Alessandria, Italy; 2 Laboratorio Integrato di Ricerca Preclinica, AOU “SS Antonio E Biagio e Cesare Arrigo”, Via Venezia 16, 15121 Alessandria, Italy

**Keywords:** Chemotherapy, GRP78, Mesothelioma, Synergy

## Abstract

Pleural mesothelioma (PM) is a rare cancer affecting the pleural layer on the body’s serosal surfaces. Exposure to asbestos fibers, a naturally occurring fibrous material with insulating characteristics, contributes to PM’s prevalence. PM has a long latency period, making major surgery ineffective and necessitating systemic treatment. Despite the progress of mesothelioma treatment, the median survival is very poor; so, there is a strong need to explore new therapeutic approaches. This study explores the use of BOLD-100, a novel therapeutic drug that targets GRP78, a protein overexpressed in PM cells. BOLD-100, a ruthenium-based small molecule therapeutic drug, is being investigated for the treatment of advanced gastrointestinal malignancies in conjunction with chemotherapy. Our aim is to investigate cellular responses of several PM cell lines to a regimen that includes BOLD-100 in addition to other commonly used treatments. BOLD-100 is a ruthenium-based anticancer therapeutic.

**Supplementary Information:**

The online version contains supplementary material available at 10.1007/s10637-025-01540-9.

## Introduction

Pleural mesothelioma (PM) is an uncommon type of cancer that mostly affects the pleural layer on the body’s serosal surfaces. A group of naturally occurring fibrous materials with outstanding physical and electrochemical insulating characteristics and a long history of industrial usage, asbestos fibers, are the major cause of PM when exposed to them in the workplace or in the environment [1]. The long incubation period (20 to 50 years) between asbestos exposure and the development of the tumor contributes to the prevalence of PM, even though the material has been gradually banned from manufacturing and use in over 50 nations worldwide since the 1980 s [2].

PM is usually discovered at a locally advanced stage because of its lengthy latency period (20 to 50 years), which renders major surgery useless and calls for systemic treatment. For around 20 years, chemotherapy (such as gemcitabine) in combination with either carboplatin or cisplatin plus pemetrexed has been the only approved first-line treatment, despite its poor response outcomes and median overall survival that is just slightly more than 12 months [3]. No meaningful treatment advance had happened until the emergence of immunotherapy, specifically immune checkpoint inhibitors (ICIs). The recent phase III trial Checkmate-743 found that the combination of two ICIs, ipilimumab and nivolumab, improved overall survival statistically significantly when compared to pemetrexed and platinum treatment (median overall survival (OS) of 18.1 versus 14.1 months, *p* = 0.002), especially for non-epithelioid subtypes [4]. So, chemotherapy with platinum compounds and pemetrexed remains the backbone of PM treatment. Immunotherapy with double immune checkpoint blockade is a major advance in PM treatment but it is not available in every country for every patient.

And there is no standard option in PM patients in further line. Gemcitabine, vinorelbine and immunotherapy could be used with underwhelming results. More prospective studies based on biomarkers are needed to identify appropriate treatment strategy [4].

Previously, our laboratory reported [5, 6] that the expression of endoplasmic reticulum (ER)-stress-related protein GRP78 (Glucose Regulated Protein 78) displayed high levels in PM cells than in mesothelial ones. In particular, we reported a differential expression among histotypes.

These findings, which show elevated basal levels of GRP78 expression in PM cells, are consistent with earlier immunohistochemical studies on biopsy tissues [5, 7, 8]. In light of this, we investigated the possible effects of BOLD-100, a ruthenium-based small drug that not only induces cell cycle arrest and DNA damage but also modifies the unfolded protein response (UPR) by selectively inhibiting GRP78, a mechanism that seems challenging to circumvent [9, 10]. After receiving BOLD-100 treatment, cells die by apoptosis due to the activation of caspases 3/7 and 8. In addition to caspases, the instability of the mitochondrial membrane triggers the BOLD-100-induced apoptotic pathway [6]. In a phase 1b/2 clinical trial, BOLD-100 is presently being investigated for the treatment of advanced gastrointestinal malignancies in conjunction with the chemotherapy FOLFOX (fluorouracil, oxaliplatin, and leucovorin) (NCT04421820; EudraCT Number: 2022–003079-41). In advanced stomach, colon and hepatobiliary cancers, preliminary data has demonstrated significant increases in Progression-Free Survival (PFS) and Overall Survival (OS) [11–14].

Therefore, the goal of this study is to examine how various PM cell lines respond to a regimen that incorporates BOLD-100 along with other frequently used PM therapy agents.

## Materials and methods

### Drugs

The inhibitor of GRP78 (sodium trans-[tetrachlorobis(1H-indazole) ruthenate(III)]) was obtained from Bold Therapeutics Inc. (Vancouver, Canada), cisplatin (cis-diamineplatinum(II) dichloride) was from Cayman Chemicals (Cabru s.a.s., Italy), gemcitabine (Gemzar) and vinorelbine (Navelbine) were from Ely Lilly Italia S.p.A. (Sesto Fiorentino, Italy).

### Cell culture and reagents

With the exception of otherwise specified, reagents were all purchased from Merck. For the experiments, the following human PM cell lines were utilized: MSTO-211H and MPP89 were obtained from ATCC (Manassas, VA, USA) [15], while four primary PM cell lines (570, 718, 729 and 748) were obtained from the Biobank of the SS. Antonio and Biagio and Cesare Arrigo University Hospital, Alessandria, Italy [16]. Of these cell lines, 718, 729, and MPP89 were epithelioid-derived; MSTO-211H is biphasic-derived, and 570 is sarcomatoid-derived. 748 histotype derivation was not available.

The complete medium (DMEM (high glucose, 4.5 g/L), 10% fetal bovine serum (FBS, Euroclone, Pero, Italy), L-glutamine (200 mM), 100 U/mL penicillin, and 100 mg/mL streptomycin was used to cultivate the cells in a humidified environment with 5% CO_2_ at 37 °C [17].

### Western blotting analysis

The Western blot procedure was followed as previously performed [18]. After being lysed in RIPA solution containing a mixture of protease and phosphatase inhibitors, the cells were solubilized in Laemmli buffer. 20 µg of proteins from cell lysates, measured by BCA Protein Assay Kit (Cayman Chemicals), were then separated using SDS–PAGE, blotted onto nitrocellulose membrane, and probed with anti-GRP78/BiP mouse monoclonal antibody (cat# 66574, RRID: AB_2881934, Proteintech, Rosemont, IL, USA). The appropriate secondary antibody (Bethyl Laboratories, Montgomery, TX, USA; dilution 1: 1000) coupled to horseradish peroxidase was subsequently incubated on the membranes using a Thermo Scientific Pierce ECL Western Blotting Substrate kit, and developed using an iBright™ CL 1500 Imaging System (Thermo-Fisher, Milano, Italy). Total protein normalization was obtained with No-Stain Protein Labeling Reagent (Thermo-Fisher) in conjunction with the iBright Imaging System.

### Cytotoxicity assay

The evaluation of single drug and drug combination cytotoxicity was carried out by using calcein-AM (calcein acetoxymethylester). Growing cells in 96-well plates were exposed to a range of doses for 48 h, then they were rinsed with PBS and incubated for 30 min at 37 °C with 2.5 μM calcein-AM in PBS. The resultant fluorescence was measured using the Infinite 200 Pro plate-reader (Tecan, Wien, Austria) at 485 nm for excitation and 535 nm for emission [6].

### Evaluation of the combined effect of drugs under fixed molar ratio

For the determination of each single drug cytotoxicity, eight doses, assessed in eightfold, between 5 and 95% cell death fractions were utilized to obtain regression estimates. The dose response sigmoidal curve fit to a two parameter logistic function of the type:

$$\text{fa}= 1 / [1+1/{(\text{D}/\text{Dm})}^{\text{m}}]$$

where D is the dose, Dm is the dose required to achieve the median cytotoxic effect, fa is the fraction of dead cells, and m is a measurement of the sigmoidicity of the curve.

After obtaining the dose–response curve for BOLD-100 and other drugs, a third experiment combining BOLD-100 with a single agent is required to evaluate whether the interaction is additive, synergic, or antagonistic. BOLD-100 and any other single drugs have different relative potencies (EC_50_s), in particular one drug is less potent than the other one in each couple. For this reason, a fixed molar ratio was used in the combination based on the relative potency EC_50_(drug1)/EC_50_(drug2).

The EC_50_ for the combination is then computed empirically using the equation:

$${\text{EC}}_{50}(\text{a}+\text{b}) = {10}^{\{[\text{log}(\text{EC}50(\text{a})) +\text{ log}(\text{EC}50(\text{b}))]/2\}}$$

The combination will then be considered as a novel drug, and higher or lower doses than the empirical EC_50_ will be tested [19].

These data were used to calculate the combination index (CI) for each cytotoxic effect level [20, 21]. The CI for each effect level indicates what type of interaction takes place between the two drugs across the entire dose range.

The CI approach estimates the whole range of cytotoxic effects using data from both single and combination dose–effect equations [19]. When the interaction is additive, CI equals 1. When CI < 1, the combination is synergic, whereas CI > 1 suggests antagonism. Synergy occurs when the actions of two drugs combine to provide a higher cytotoxic effect, while antagonism results in a reduced cytotoxic effect compared to the additive effects of single drugs.

### JC-1 assay for mitochondrial membrane potential

The Infinite 200 Pro well plate reader (Tecan, Vienna, Austria) was used to measure the bulk red and green fluorescence from cells tagged with JC-1 in 96-well plates. In order to determine the mitochondrial membrane potential, red (ex = 535, em = 590 nm) and green (ex = 485, em = 535) fluorescence values were measured. Red/green ratios were then computed [6].

### Statistical analysis

Statistical analysis was carried out with GraphPad Prism 8 (Graphpad Software Inc, GraphPad Software, Inc, San Diego, CA, USA). The figure legends for each experiment provide statistical information (test used, value of *n*, replicates, *p* value, etc.).

## Results

### GRP78 expression

**Fig. 1:**
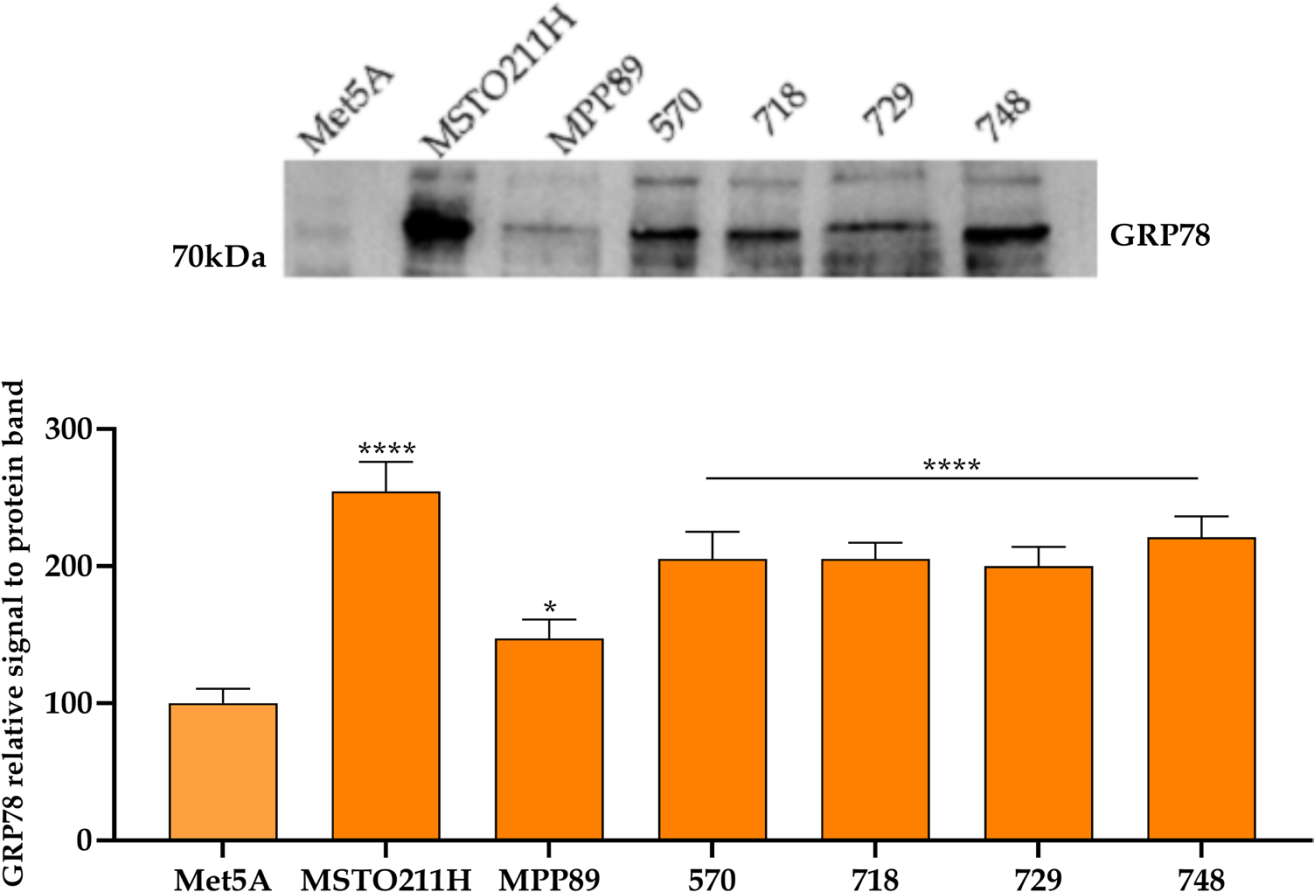
GRP78 protein expression in mesothelial Met5 A cells compared to pleural mesothelioma cells*.* Blots in the upper part of the figure are representative of three; each lane was loaded with 20 μg of proteins, probed with anti-GRP78 mouse mouse-clonal antibody and managed as described in the “Materials and methods” section. Bars in the lower part of the figure are means ± SD derived from three independent samples. Statistics indicate differences between Met5A and PM cell lines (* *p* < 0.05, **** *p* < 0.0001; one-way ANOVA followed by Dunnet post-test)

BOLD-100 [6, 22, 23] acts through a selective GRP78 inhibition displaying a more toxic behavior on PM cells than on non-cancerous mesothelial cells. Therefore, by using Western blotting analysis, we evaluated basal expression of GRP78 in the cell lines we chose to utilize in order to determine whether treatment with BOLD-100 was feasible. As illustrated in Fig. 1, the expression of GRP78 is significantly greater in all the mesothelioma cell lines analyzed compared to the mesothelial non-cancerous Met5A. However, the expression in the MPP89 line is marginally higher than the baseline level of mesothelial cells.

### Cells viability assay

After a 48-h treatment with single drug, PM cells showed a dose-dependent effect with EC_50_ values as in Table 1. As demonstrated before [6], the EC_50_ value for Met5 A cells is 399 µM. As we previously showed [6] in an epithelioid PM cell line, BOLD-100 reduces the surviving factor by half. The result has now been confirmed in a biphasic PM cell line (MSTO-211H). After treatment with a single chemotherapeutic, we also assessed the surviving factor percentage, and all of them cut it in half as compared to the control. The possibility of BOLD-100 + single drug combinations is evaluated, and a further significant decrease is observed. It is specifically decreased by roughly 90%, 85%, and 95% following treatment with BOLD-100 + Gem, BOLD-100 + cisPt, and BOLD-100 + Vin, respectively (Supplementary material 1).

**Table 1:** Cell viability after single drug treatment expressed as EC_50_ values. Drug concentrations are indicated as µM. In brackets the 95% confidence interval. Each value is derived from 3 independent experiments

*Cell line*	*BOLD-100*	*Cisplatin* *(cisPt)*	*Gemcitabine (Gem)*	*Vinorelbine (Vin)*
*MSTO211H*	129.01 (76.37–217.95)	31.75 (24.63–40.91)	10.05 (6.80–14.69)	2.79 (1.65–4.70)
*MPP89*	78.89 (64.56–96.38)	14.31 (9.29–22.03)	22.04 (14.73–32.96)	8.33 (3.20–21.65)
*570*	46.80 (39.91–54.76)	22.28 (14.43–34.49)	7.21 (3.80–13.46)	2.03 (1.03–3.99)
*718*	54.30 (46.34–63.63)	78.24 (65.70–93.18)	10.42 (4.31–25.05)	1.94 (0.69–5.45)
*729*	142.24 (93.55–216.23)	54.82 (35.78–83.90)	44.26 (23.70–82.65)	0.34 (0.07–1.69)
*748*	113.35 (59.83–214.75)	70.53 (40.31–123.38)	37.49 (27.36–51.35)	3.38 (0.83–13.63)

**Fig. 2:**
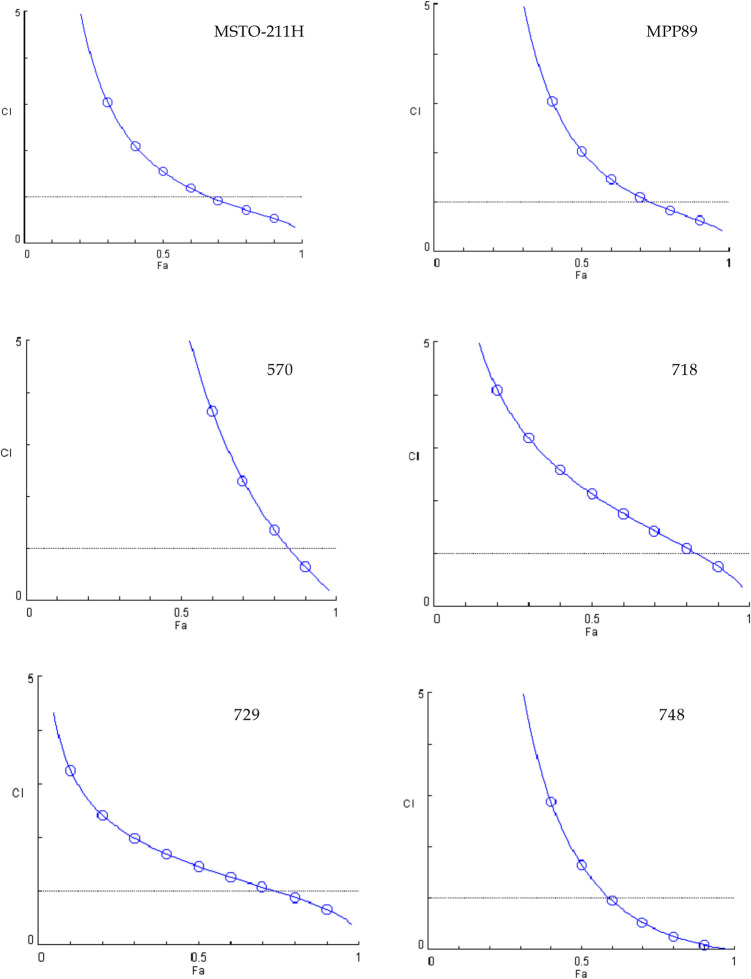
Combination index plots (fa–CI plots) for BOLD-100/cisplatin combination*.* CI values are plotted against the fractional inhibition (fa) of MSTO-211H, MPP89, 570, 718, 729 and 748 cell viability, as obtained by the Calcein-AM endpoint. CI < 1 indicates synergy, CI = 1 indicates additive effect, and CI > 1 indicates antagonism. Six replicates in three independent experiments were used

**Fig. 3:**
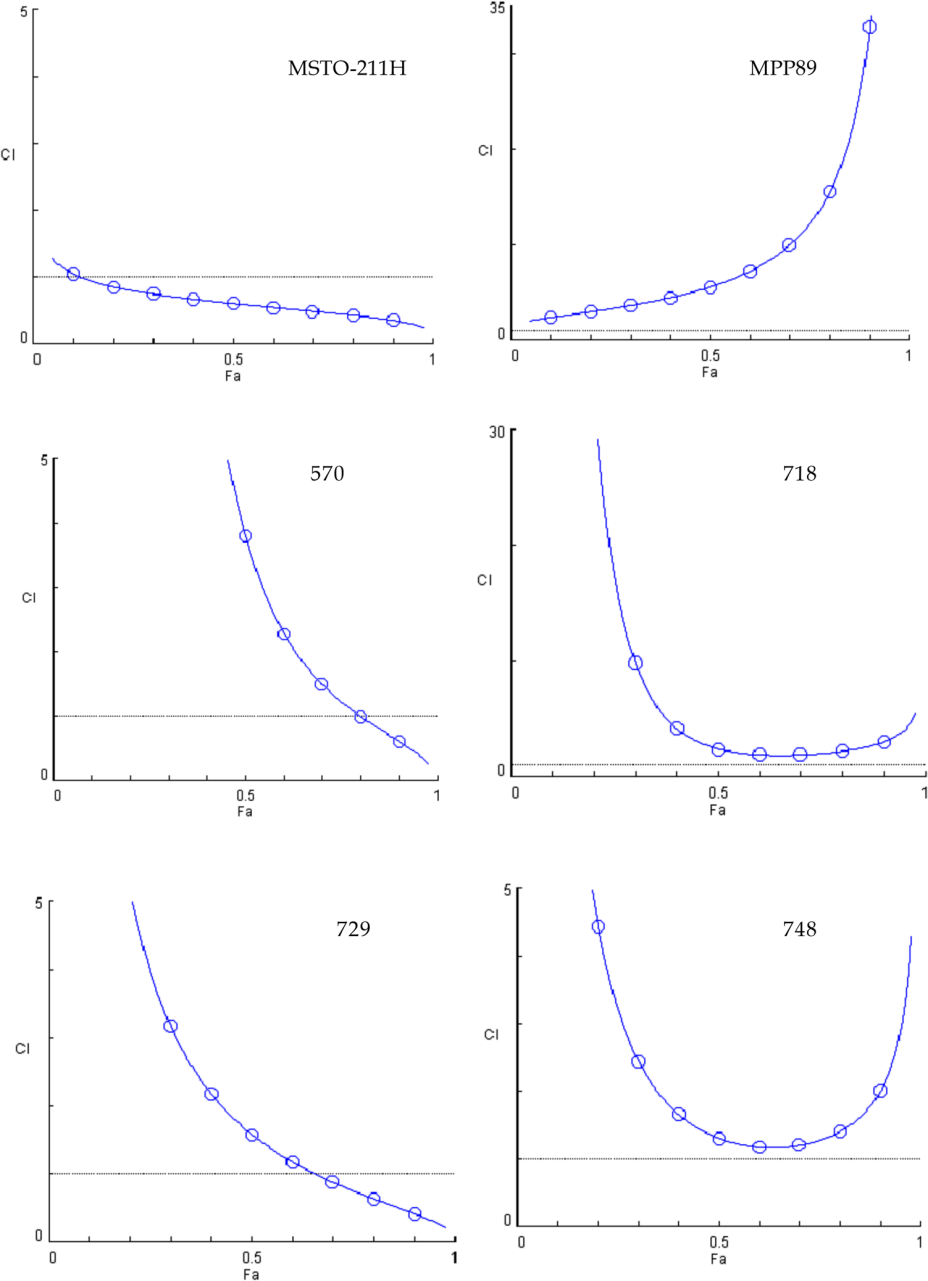
Combination index plots (fa–CI plots) for BOLD-100/gemcitabine combination*.* CI values are plotted against the fractional inhibition (fa) of MSTO-211H, MPP89, 570, 718, 729 and 748 cell viability, as obtained by the Calcein-AM endpoint. CI < 1 indicates synergy, CI = 1 indicates additive effect, and CI > 1 indicates antagonism. Six replicates in three independent experiments were used

**Fig. 4:**
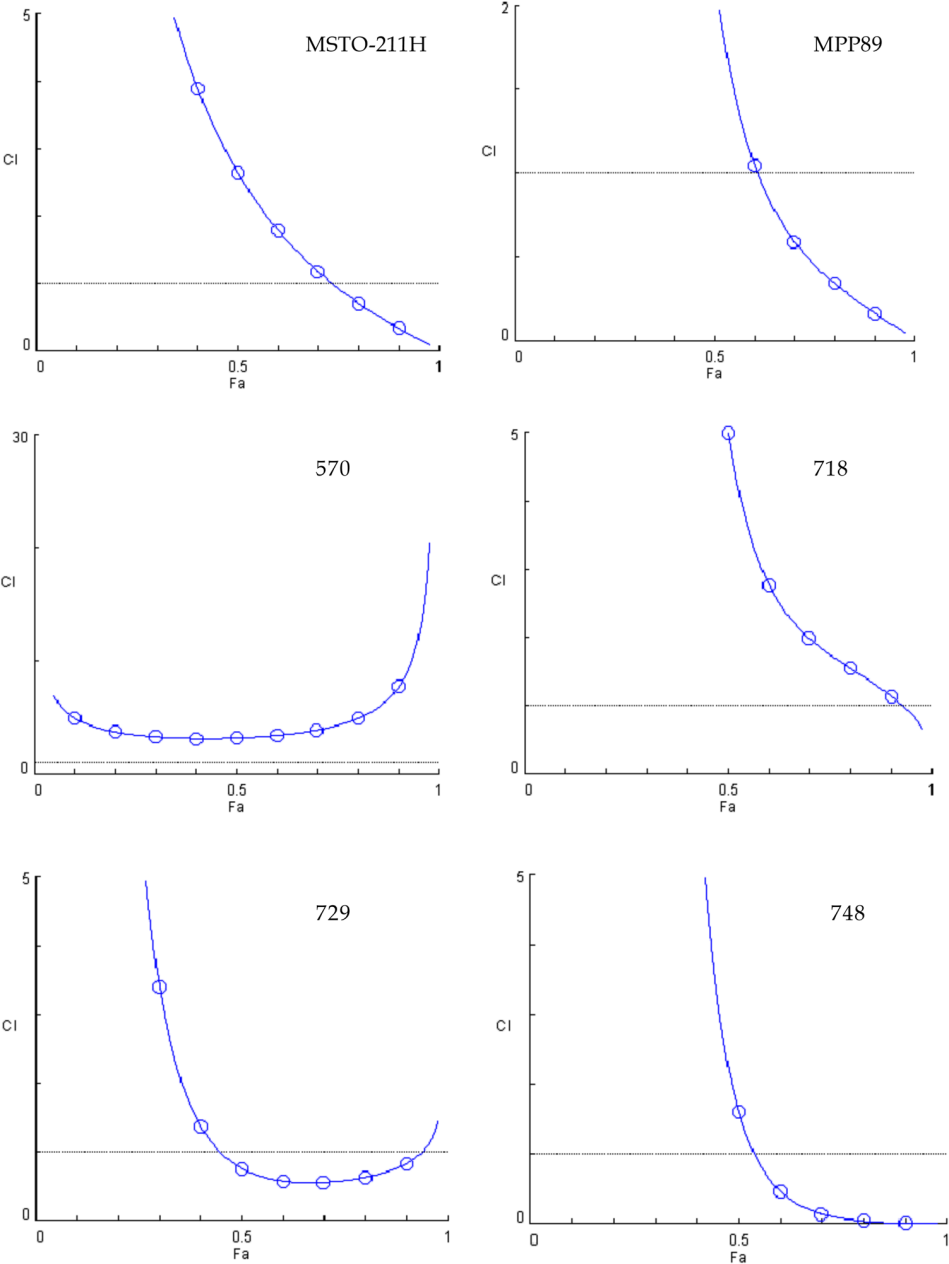
Combination index plots (fa–CI plots) for BOLD-100/vinorelbine combination. CI values are plotted against the fractional inhibition (fa) of MSTO-211H, MPP89, 570, 718, 729 and 748 cell viability, as obtained by the Calcein-AM endpoint. CI < 1 indicates synergy, CI = 1 indicates additive effect, and CI > 1 indicates antagonism. Six replicates in three independent experiments were used

After combining every drug with BOLD-100 for any given cell line, the combination index (CI) was computed pointing to synergistic, additive, or antagonistic behavior at all the affected fractions (fa) in a range of 0.1 to 0.9. Figures 2, 3, and 4 show the behavior of the distinct cell lines in response to the treatments. All of the lines studied, despite varying trends and levels, demonstrated synergistic behavior at the effect level of 0.9 following treatment with the BOLD-100 + cisplatin combo.

In contrast, when treated with the BOLD-100 + gemcitabine combination, only three out of six lines, specifically MSTO-211H, 570, and 729, demonstrated synergistic behavior at fa 0.9, with the MSTO-211H line exhibiting synergistic behavior at practically all effect levels. For this combination, the MPP89, 718, and 748 lines, while nearing additivity, have never showed synergistic behavior, and the MPP89 line exhibits notable antagonistic behavior as the effects grow.

Four of the six lines exhibit synergistic action when BOLD-100 and vinorelbine are combined. Line 570, in instance, never approaches the threshold line, indicating additivity, whereas line 718 achieves additivity at the fa0.9 effect level before descending below the threshold line.

To highlight the shifts in the behavior of the three combinations toward the cell lines, we concentrated on three different impact levels, fa 0.5—0.9—0.97, which represent the most intriguing effect levels in oncology therapy [24].

**Fig. 5:**
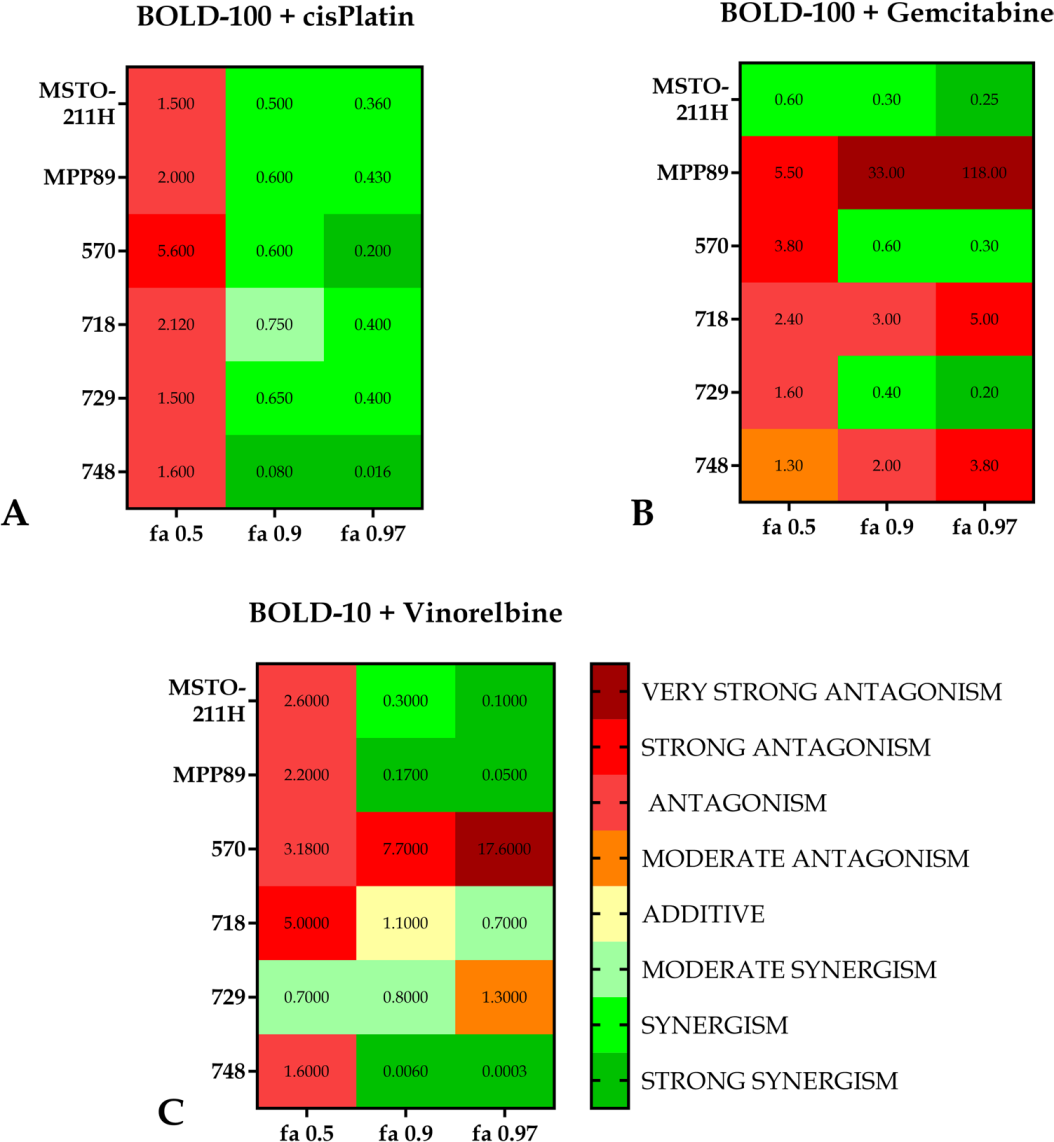
Heat maps for combination of BOLD-100/drug at different fa. Heat map showing cell lines and fractional inhibition (fa) at value 0.5, 0.9, 0.97. The entries are CI values. The CIs are mapped as 0–0.29 strong synergism, 0.3–0.69 synergism, 0.7–0.89 moderate synergism, 0.9–1.1 additive, 1.11–1.3 moderate antagonism, 1.31–3.3 antagonism, 3.31–10 strong antagonism, 10.1- ∞ very strong antagonism (Modified from Chou, 2006 [40]). Panel **A** is referred to BOLD-100+cisPlatin combination, Panel **B** to BOLD-100+Gemcitabine, Panel **C** to BOLD-100+Vinorelbine

Figure 5 shows that at level fa 0.5, only two CI values are less than 1. Considering effects levels fa 0.9 and fa 0.97, the bulk of the observed CI values are less than one, and they decrease when the effect level is increased. Only in four cases that the results do not decrease; as the effect rises, they approach values indicating significant antagonism. The combination with the most promising outcomes is BOLD-100 + cisPt, whereas the least promising is BOLD-100 + Gem.

### Mitochondrial membrane potential assay

An important part of the energy storage process during oxidative phosphorylation is the mitochondrial membrane potential (ΔΨm), which is produced by proton pumps (Complexes I, III, and IV). The transmembrane potential of hydrogen ions, which is used to create ATP, is formed by ΔΨm and the proton gradient (ΔpH). Although there are occasional variations in both ATP and ΔΨm, which are indicative of normal physiological activity, the amounts of these substances in the cell are maintained at a generally constant level. Long-term alterations in these variables, however, could be harmful. A persistent decrease or increase in ΔΨm relative to normal levels can lead to a number of diseases and unintended cell viability loss [25]. Previous work from our laboratory [6] shows, by staining cells with JC-1 probe, that BOLD-100 destabilizes mitochondrial membrane potential, and literature suggests that the drugs used in the combination also cause mitochondrial damage [26–28].

**Fig. 6:**
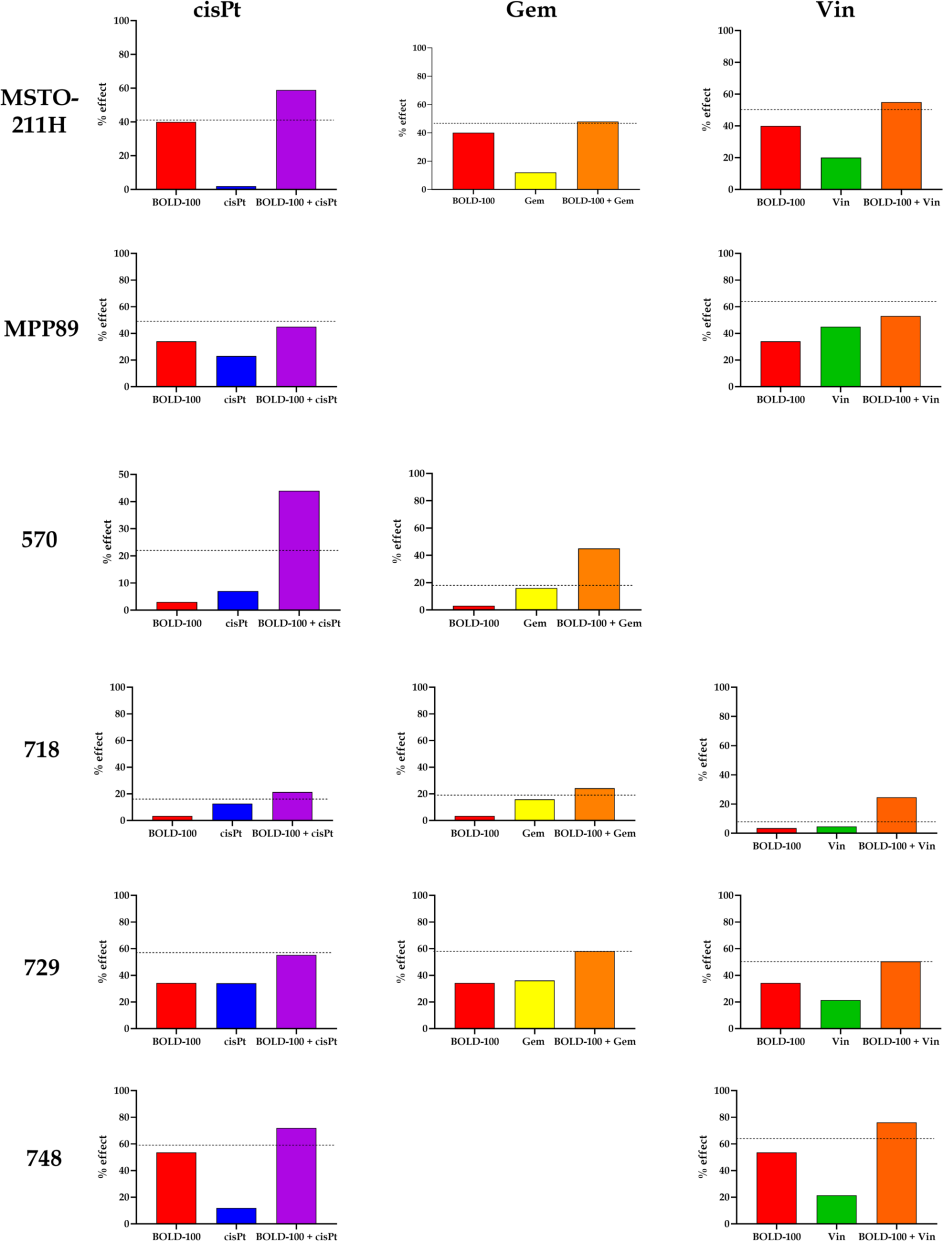
Mitochondrial membrane potential. Mitochondrial membrane potential (ΔΨm) variation after 1-h exposure to single agents (BOLD-100 and single drugs) or combinations BOLD-100 + drug. Data resulting from 5 independent treatments have been obtained as JC-1 red/green fluorescence ratio and converted as % of effect on mitochondrial membrane potential stability (so, the control condition, not indicated, is posed at zero). In the graphs, the % of effect is referred to the expected effect indicated as a dotted line

To assess the synergistic activity of combinations, the ΔΨm was examined using a functional test. Cell lines and combinations that did not demonstrate additivity or synergy at any effect level were disregarded. It was chosen, in particular, to assess the effect of treatment with the drugs alone or in combination at a concentration equivalent to the EC_50_. The experimental data were then compared to the expected results calculated using the Bliss independence model (Fig. 6).

**Fig. 7:**
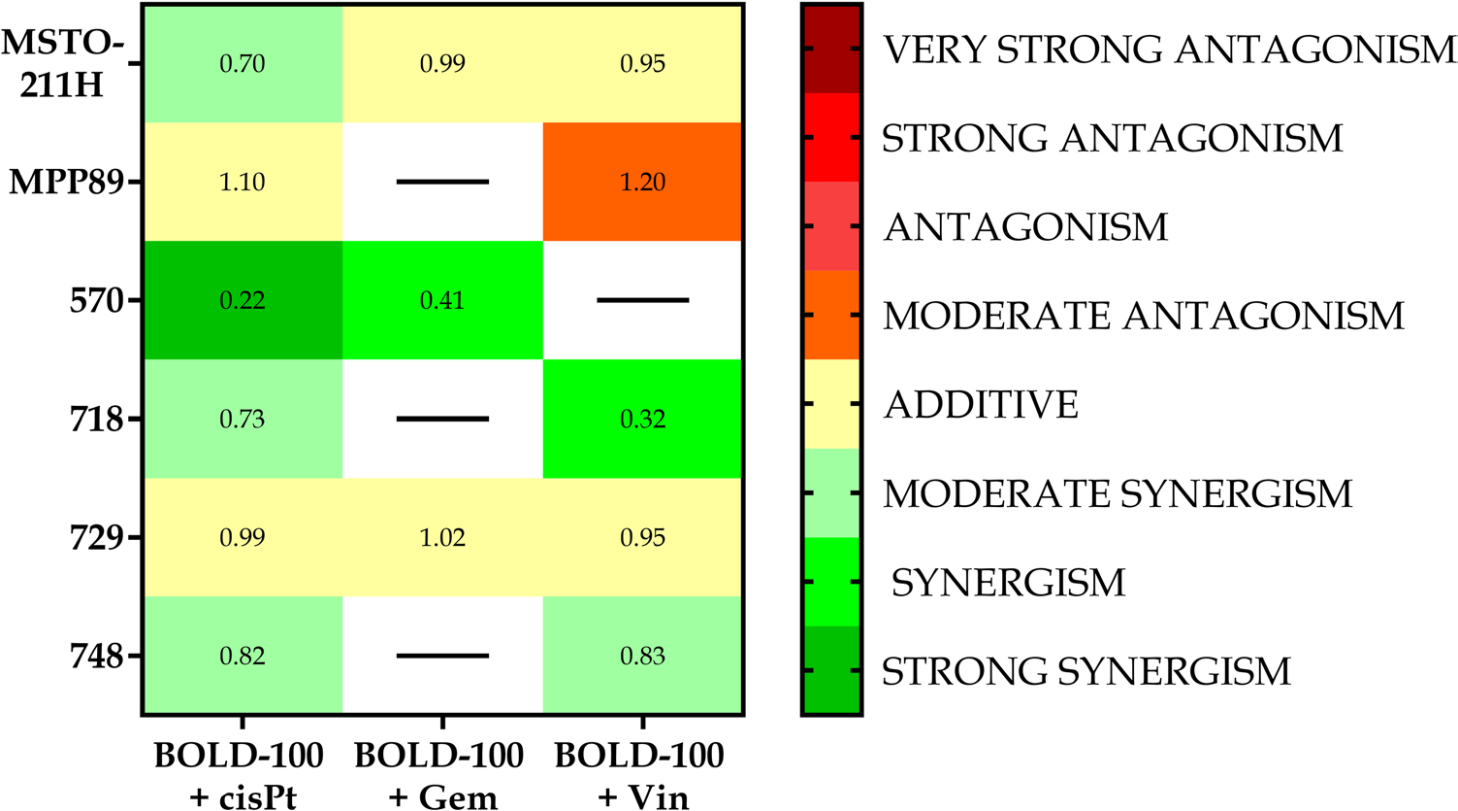
Heat maps for combination of BOLD-100/drug at fa 0.5. Heat map showing cell lines and combinations BOLD-100/drug. The entries are CI values obtained from ΔΨm data. The CIs are mapped as 0–0.29 strong synergism, 0.3–0.69 synergism, 0.7–0.89 moderate synergism, 0.9–1.1 additive, 1.11–1.3 moderate antagonism, 1.31–3.3 antagonism, 3.31–10 strong antagonism, 10.1- ∞ very strong antagonism (Modified from Chou, 2006 [40])

To better define the behavior of combination of BOLD-100 and drugs, the ratio of expected effect to observed effect was evaluated to get the CI value. When comparing the data on cell viability at the fa0.5 effect level shown in the earlier heat maps (Fig. 5) with the data on the stability of the mitochondrial membrane potential shown in Fig. 7, we find that, when taking a more functional approach and using the Bliss independence model, there is seldom any significant antagonistic behavior and, instead, a synergistic behavior.

## Discussion

When compared to monotherapy regimens, combination chemotherapy can prolong the management of many incurable cancers and is commonly utilized to cure specific cancer types. Because the likelihood of developing resistance to multiple drugs with distinct mechanisms is lower than that of developing resistance to a single drug, combining multiple, independently effective chemotherapeutic mechanisms could overcome tumor heterogeneity and lead to more patients experiencing longer-lasting remissions, if not cures [29, 30].

In addition to inducing the expression and activation of pro-survival molecules like GADD34 and GRP78, ER stress can also activate pro-apoptotic molecules such as CHOP (C/EBP homologous protein) and caspase-12. The equilibrium between these processes dictates the fate of the cell, which can either be adaptation or apoptosis. Therefore, the development of a resistant phenotype is determined by the tumor cells'tolerance to endoplasmic reticulum stress, which they achieve by preventing cell death by apoptosis [31]. There are many examples in the literature of anticancer drug combinations with chemicals that can exacerbate ER stress and upset the balance in favor of apoptosis [31–33].

As we previously demonstrated, BOLD-100 induces damage to cancer cells through a variety of methods, including apoptosis. BOLD-100 suppresses GRP78 and modifies the unfolded protein response (UPR), while also increasing reactive oxygen species (ROS), which causes DNA damage [22, 34]. This ROS production, comparable to that determined by standard chemotherapy drugs, is significantly magnified when BOLD-100 is used in combination with the individual drugs (Supplementary material 2). BOLD-100 can then increase cancer cell death by synergizing with cytotoxic chemotherapies and targeted medicines.

This study investigated three drugs that are currently used in the treatment of pleural mesothelioma: cisplatin, presently used as first-line systemic therapy in combination with pemetrexed [35, 36], gemcitabine and vinorelbine, used in second-line treatment [36], in combination with BOLD-100, that is currently tested in clinical trial in combination with oxaliplatin and fluorouracil [11, 12].

By binding to DNA and creating intra-strand DNA adducts, which stop DNA synthesis and cell division, cisplatin interacts with cellular macromolecules and causes cytotoxicity through a number of biochemical processes [37]. The antimetabolite gemcitabine, as nucleoside analog, inhibits tumor growth by converting into active triphosphorylated nucleotides that interfere with DNA synthesis and target ribonucleotide reductase [38]. Vinorelbine is an antimitotic anticancer drug whose primary mode of action is the suppression of microtubule dynamics, resulting in mitotic arrest and cell death [39].

The four mechanisms of action eventually lead to cell death by apoptosis, generating several signal cascades that, in three out of four situations, share DNA as a common target. Nevertheless, given that the main effect of BOLD-100 is on the activity of GRP78 and the UPR, we decided to analyze the synergy between the drugs and BOLD-100 using the combination index based on the Chou-Talalay model, which predicts an interaction between the two components of the mixture, and the Bliss independence model, in which the two components do not interact and act on two different targets [40]. After acquiring the dose–response curve for BOLD-100 and other drugs, a third experiment was specifically designed to assess if the interactions between BOLD-100 and the individual drugs were antagonistic, synergistic, or additive. A fixed molar ratio based on the respective potencies of drugs 1 and 2 was employed to determine the mixture’s therapeutic concentrations since in each pair, one of them is less potent than the other. As a result, a range of concentrations above and below the empirically determined EC_50_ will be evaluated, and the combination will be regarded as a novel drug [19].

Viability assessment following cytotoxic drug exposure fails to evaluate the cell death phenotype, hence drug treatment can induce any form of cell death phenotype, including passive necrosis [41]. Beginning with the idea that BOLD-100’s mode of action is able to disrupt the mitochondrial membrane potential (ΔΨm) [6], we also assessed whether the combination worked in concert to cause this event and the ensuing cell death.

To have a descriptive pool of cell lines representing the histological variability that characterizes PM, we used a line with a biphasic histotype (MSTO211H), one with a sarcomatoid histotype (570), three with an epithelioid histotype (MPP89, 718, 729) and one with an unavailable histotype (748). With particular reference to GRP78 expression, the variation in the protein's baseline between cell lines is visible.

When examining the combination indexes (CI) obtained from data gained with cell viability assay after treatment with the three combinations studied, it becomes clear that the combination with cisplatin is the most promising because a synergistic impact was produced for all of the cell lines that were considered, at least for the highest effect values.

On the other hand, gemcitabine is the least effective combination; in fact, only three out of the six lines showed a synergistic effect. Additionally, it is clear from comparing the cell lines that, when taking into account both the mitochondrial membrane potential assay and cell viability, MSTO211H were the most responsive to the treatments given utilizing the combinations. However, MPP89 cells showed the worst outcomes, which was in line with their reduced GRP78 expression. The sarcomatoid histotype, represented by line 570, offers more pertinent details.

Typically, cancers with this histotype have a more advanced start of resistance. A synergistic behavior was demonstrated by the two combinations, BOLD-100 + cisPt and BOLD-100 + Gem.

At the present, these data are not enough for a translational evidence regarding GRP78 as a biomarker in PM, but a greater knowledge on the role of this protein could be very useful in the evaluation of PM. However, multi-drug approach, with BOLD-100 as combination drug, can significantly increase the efficacy of methods currently employed for PM therapy.

This work establishes the groundwork for a more comprehensive exploration of GRP78 expression and tissue location in PM, its association with the emergence of resistance phenotype, and its potential utility as an extra therapeutic target. For the treatment of advanced gastrointestinal malignancies, BOLD-100 is presently undergoing phase 2 clinical development. We now showed that BOLD-100, when combined with anticancer drugs, showed synergistic behavior in some PM cell lines, especially in treatment-resistant cell lines. Our data clearly emphasizes the necessity for a clinical trial to assess the effectiveness of BOLD-100 in the PM population, given these findings and the dearth of viable therapies for PM.

## Supplementary Information

Below is the link to the electronic supplementary material.

Supplementary file1 (PDF 718 KB)

## Data Availability

No datasets were generated or analysed during the current study.

## References

[1] Duncan LT (2021) Advances in health and disease, vol 44. Nova Publishers Inc., Hauppauge, New York. 10.52305/FRKL1404

[2] Cao C, Croce B, Harris R (2012) MPM: malignant pleural mesothelioma. Ann Cardiothorac Surg 1(4):544. 10.3978/j.issn.2225-319X.2012.11.03 23977552 10.3978/j.issn.2225-319X.2012.11.03 PMC3741783

[3] Santoro A, O’Brien ME, Stahel RA, Nackaerts K, Baas P, Karthaus M et al (2008) Pemetrexed plus cisplatin or pemetrexed plus carboplatin for chemonaïve patients with malignant pleural mesothelioma: results of the international expanded access program. J Thorac Oncol 3(7):756–763. 10.1097/JTO.0b013e31817c73d6 18594322 10.1097/JTO.0b013e31817c73d6

[4] Baas P, Scherpereel A, Nowak AK, Fujimoto N, Peters S, Tsao AS et al (2021) First-line nivolumab plus ipilimumab in unresectable malignant pleural mesothelioma (CheckMate 743): a multicentre, randomised, open-label, phase 3 trial. Lancet 397(10272):375–386. 10.1016/S0140-6736(20)32714-8 33485464 10.1016/S0140-6736(20)32714-8

[5] Martinotti S, Ranzato E, Burlando B (2018) (-)- Epigallocatechin-3-gallate induces GRP78 accumulation in the ER and shifts mesothelioma constitutive UPR into proapoptotic ER stress. J Cell Physiol 233(10):7082–7090. 10.1002/jcp.26631 29744892 10.1002/jcp.26631

[6] Ranzato E, Bonsignore G, Martinotti S (2022) ER Stress response and induction of apoptosis in malignant pleural mesothelioma: the achilles heel targeted by the anticancer ruthenium drug BOLD-100. Cancers (Basel) 14(17). 10.3390/cancers14174126 10.3390/cancers14174126 PMC9454852 36077664

[7] Manfredi M, Martinotti S, Gosetti F, Ranzato E, Marengo E (2016) The secretome signature of malignant mesothelioma cell lines. J Proteomics 145:3–10. 10.1016/j.jprot.2016.02.021 26921831 10.1016/j.jprot.2016.02.021

[8] Dalton LE, Clarke HJ, Knight J, Lawson MH, Wason J, Lomas DA et al (2013) The endoplasmic reticulum stress marker CHOP predicts survival in malignant mesothelioma. Br J Cancer 108(6):1340–1347. 10.1038/bjc.2013.66 23412101 10.1038/bjc.2013.66 PMC3619254

[9] Burris HA, Bakewell S, Bendell JC, Infante J, Jones SF, Spigel DR et al (2016) Safety and activity of IT-139, a ruthenium-based compound, in patients with advanced solid tumours: a first-in-human, open-label, dose-escalation phase I study with expansion cohort. ESMO Open 1(6):e000154. 10.1136/esmoopen-2016-000154 28848672 10.1136/esmoopen-2016-000154 PMC5548977

[10] Griffin D, Carson R, Moss D, Sessler T, Lavin D, Tiwari VK et al (2024) Ruthenium drug BOLD-100 regulates BRAFMT colorectal cancer cell apoptosis through AhR/ROS/ATR signaling axis modulation. Mol Cancer Res. 10.1158/1541-7786.MCR-24-0151 10.1158/1541-7786.MCR-24-0151 PMC7616621 39083088

[11] Spratlin JL, O’Kane G, Goodwin RA, McWhirter E, Thompson D, Halani K et al (2022) BOLD-100–001 (TRIO039): a phase 1b dose-escalation study of BOLD-100 in combination with FOLFOX chemotherapy in patients with advanced gastrointestinal solid cancers: interim safety, tolerability, and efficacy. J Clin Oncol 40(16). 10.1200/JCO.2022.40.16_suppl.303

[12] O’Kane GM, Spratlin JL, Oh D-Y, Rha SY, Elimova E, Kavan P et al (2023) BOLD-100-001 (TRIO039): a phase 1b/2a study of BOLD-100 in combination with FOLFOX chemotherapy in patients with pre-treated advanced gastric and biliary tract cancer: efficacy and safety analysis. J Clin Oncol 41(16_suppl). 10.1200/JCO.2023.41.16_suppl.4098

[13] O’Kane GM, Oh D-Y, Spratlin J, Rha SY, Elimova E et al (2024) A phase 2 study of BOLD-100 in combination with FOLFOX chemotherapy in patients with pretreated advanced biliary tract cancer: efficacy and safety analysis (BOLD-100–001). J Clin Oncol 42. 10.1200/JCO.2024.42.16_suppl.4115

[14] Spratlin JL, O’Kane GM, Oh D-Y, Rha SY, McWhirter E, Elimova E et al (2024) A phase 2 study of BOLD-100 in combination with FOLFOX in patients with advanced mCRC previously treated with FOLFOX/CAPOX—efficacy and safety analysis. J Clin Oncol 43. 10.1200/JCO.2024.42.3_suppl.143

[15] Napoli F, Rapa I, Izzo S, Rigutto A, Libener R, Riganti C et al (2022) Micro-RNA-215 and -375 regulate thymidylate synthase protein expression in pleural mesothelioma and mediate epithelial to mesenchymal transition. Virchows Arch 481(2):233–244. 10.1007/s00428-022-03321-8 35461395 10.1007/s00428-022-03321-8 PMC9343276

[16] Bonvicini P, Libener R, Amore V, Oliveri G, Maconi A (2022) Alessandria Biobank: origins, evolution and future scenarios. Work Pap Public Health 10(1). 10.4081/wpph.2022.9522

[17] Ranzato E, Martinotti S, Magnelli V, Murer B, Biffo S, Mutti L et al (2012) Epigallocatechin-3-gallate induces mesothelioma cell death via H2 O2 -dependent T-type Ca2+ channel opening. J Cell Mol Med 16(11):2667–2678. 10.1111/j.1582-4934.2012.01584.x 22564432 10.1111/j.1582-4934.2012.01584.x PMC4118235

[18] Pellavio G, Martinotti S, Patrone M, Ranzato E, Laforenza U (2022) Aquaporin-6 may increase the resistance to oxidative stress of malignant pleural mesothelioma cells. Cells 11(12). 10.3390/cells11121892 10.3390/cells11121892 PMC9221246 35741021

[19] Lombardo T, Anaya A, Kornblihtt L, Blanco G (2012) Median effect dose and combination index analysis of cytotoxic drugs using flow cytometry. In: Schmid I (ed) Flow cytometry - recent perspectives. IntechOpen. London, pp 393–420. 10.5772/38214

[20] Chou TC (2008) Preclinical versus clinical drug combination studies. Leuk Lymphoma 49(11):2059–2080. 10.1080/10428190802353591 19021049 10.1080/10428190802353591

[21] Chou TC (2010) Drug combination studies and their synergy quantification using the Chou-Talalay method. Cancer Res 70(2):440–446. 10.1158/0008-5472.CAN-09-1947 20068163 10.1158/0008-5472.CAN-09-1947

[22] Bakewell S, Conde I, Fallah Y, McCoy M, Jin L, Shajahan-Haq AN (2020) Inhibition of DNA repair pathways and induction of ROS are potential mechanisms of action of the small molecule inhibitor BOLD-100 in breast cancer. Cancers (Basel) 12(9). 10.3390/cancers12092647 10.3390/cancers12092647 PMC7563761 32947941

[23] Neuditschko B, Legin AA, Baier D, Schintlmeister A, Reipert S, Wagner M et al (2021) Interaction with ribosomal proteins accompanies stress induction of the anticancer metallodrug BOLD-100/KP1339 in the endoplasmic reticulum. Angew Chem Int Ed Engl 60(10):5063–5068. 10.1002/anie.202015962 33369073 10.1002/anie.202015962 PMC7986094

[24] Wang S, Zhang H, Cheng L, Evans C, Pan CX (2010) Analysis of the cytotoxic activity of carboplatin and gemcitabine combination. Anticancer Res 30(11):4573–4578 21115908 PMC4562399

[25] Zorova LD, Popkov VA, Plotnikov EY, Silachev DN, Pevzner IB, Jankauskas SS et al (2018) Mitochondrial membrane potential. Anal Biochem 552:50–59. 10.1016/j.ab.2017.07.009 28711444 10.1016/j.ab.2017.07.009 PMC5792320

[26] Martins NM, Santos NA, Curti C, Bianchi ML, Santos AC (2008) Cisplatin induces mitochondrial oxidative stress with resultant energetic metabolism impairment, membrane rigidification and apoptosis in rat liver. J Appl Toxicol 28(3):337–344. 10.1002/jat.1284 17604343 10.1002/jat.1284

[27] Inamura A, Muraoka-Hirayama S, Sakurai K (2019) Loss of mitochondrial DNA by gemcitabine triggers mitophagy and cell death. Biol Pharm Bull 42(12):1977–1987. 10.1248/bpb.b19-00312 31787713 10.1248/bpb.b19-00312

[28] Liu Z, Fu Q, Wang Y, Cui L, Zhang W, Teng Y et al (2021) Synergy between vinorelbine and afatinib in the inhibition of non-small cell lung cancer progression by EGFR and p53 signaling pathways. Biomed Pharmacother 134:111144. 10.1016/j.biopha.2020.111144 33360044 10.1016/j.biopha.2020.111144

[29] Pomeroy AE, Schmidt EV, Sorger PK, Palmer AC (2022) Drug independence and the curability of cancer by combination chemotherapy. Trends Cancer 8(11):915–929. 10.1016/j.trecan.2022.06.009 35842290 10.1016/j.trecan.2022.06.009 PMC9588605

[30] Pritchard JR, Lauffenburger DA, Hemann MT (2012) Understanding resistance to combination chemotherapy. Drug Resist Updat 15(5–6):249–257. 10.1016/j.drup.2012.10.003 23164555 10.1016/j.drup.2012.10.003 PMC3975170

[31] Xu Y, Wang C, Su J, Xie Q, Ma L, Zeng L et al (2015) Tolerance to endoplasmic reticulum stress mediates cisplatin resistance in human ovarian cancer cells by maintaining endoplasmic reticulum and mitochondrial homeostasis. Oncol Rep 34(6):3051–3060. 10.3892/or.2015.4283 26398138 10.3892/or.2015.4283

[32] Pongking T, Thongpon P, Intuyod K, Klungsaeng S, Thanan R, Chaidee A et al (2024) Cannabidiol exhibits potent anti-cancer activity against gemcitabine-resistant cholangiocarcinoma via ER-stress induction in vitro and in vivo. BMC Complement Med Ther 24(1):325. 10.1186/s12906-024-04610-2 39215312 10.1186/s12906-024-04610-2 PMC11365133

[33] Luo C, Wang H, Liu Q (2019) A genetically encoded ratiometric calcium sensor enables quantitative measurement of the local calcium microdomain in the endoplasmic reticulum. Biophys Rep 5(31):42. 10.1007/s41048-019-0082-6

[34] Bakewell SJ, Rangel DF, Ha DP, Sethuraman J, Crouse R, Hadley E et al (2018) Suppression of stress induction of the 78-kilodalton glucose regulated protein (GRP78) in cancer by IT-139, an anti-tumor ruthenium small molecule inhibitor. Oncotarget 9(51):29698–29714. 10.18632/oncotarget.25679 30038714 10.18632/oncotarget.25679 PMC6049868

[35] Vogelzang NJ, Rusthoven JJ, Symanowski J, Denham C, Kaukel E, Ruffie P et al (2003) Phase III study of pemetrexed in combination with cisplatin versus cisplatin alone in patients with malignant pleural mesothelioma. J Clin Oncol 21(14):2636–2644. 10.1200/JCO.2003.11.136 12860938 10.1200/JCO.2003.11.136

[36] Popat S, Baas P, Faivre-Finn C, Girard N, Nicholson AG, Nowak AK et al (2022) Malignant pleural mesothelioma: ESMO Clinical Practice Guidelines for diagnosis, treatment and follow-up. Ann Oncol 33(2):129–142. 10.1016/j.annonc.2021.11.005 34861373 10.1016/j.annonc.2021.11.005

[37] Tchounwou PB, Dasari S, Noubissi FK, Ray P, Kumar S (2021) Advances in our understanding of the molecular mechanisms of action of cisplatin in cancer therapy. J Exp Pharmacol 13:303–328. 10.2147/JEP.S267383 33776489 10.2147/JEP.S267383 PMC7987268

[38] Ciccolini J, Serdjebi C, Peters GJ, Giovannetti E (2016) Pharmacokinetics and pharmacogenetics of Gemcitabine as a mainstay in adult and pediatric oncology: an EORTC-PAMM perspective. Cancer Chemother Pharmacol 78(1):1–12. 10.1007/s00280-016-3003-0 27007129 10.1007/s00280-016-3003-0 PMC4921117

[39] Capasso A (2012) Vinorelbine in cancer therapy. Curr Drug Targets 13(8):1065–1071. 10.2174/138945012802009017 22594474 10.2174/138945012802009017

[40] Chou TC (2006) Theoretical basis, experimental design, and computerized simulation of synergism and antagonism in drug combination studies. Pharmacol Rev 58(3):621–681. 10.1124/pr.58.3.10 16968952 10.1124/pr.58.3.10

[41] Healy E, Dempsey M, Lally C, Ryan MP (1998) Apoptosis and necrosis: mechanisms of cell death induced by cyclosporine A in a renal proximal tubular cell line. Kidney Int 54(6):1955–1966. 10.1046/j.1523-1755.1998.00202.x 9853260 10.1046/j.1523-1755.1998.00202.x

