# Supplementary material for: Unraveling BOLD-100 synergistic potential in pleural mesothelioma treatment: an in vitro study
Source: Invest New Drugs. 2025 May 8;43(3):634–45. doi: 10.1007/s10637-025-01540-9 (PMC12310764; doi:10.1007/s10637-025-01540-9)
Supplement: Supplementary file 1 — Supplementary file1 (PDF 718 KB) [file 10637_2025_1540_MOESM1_ESM.pdf]

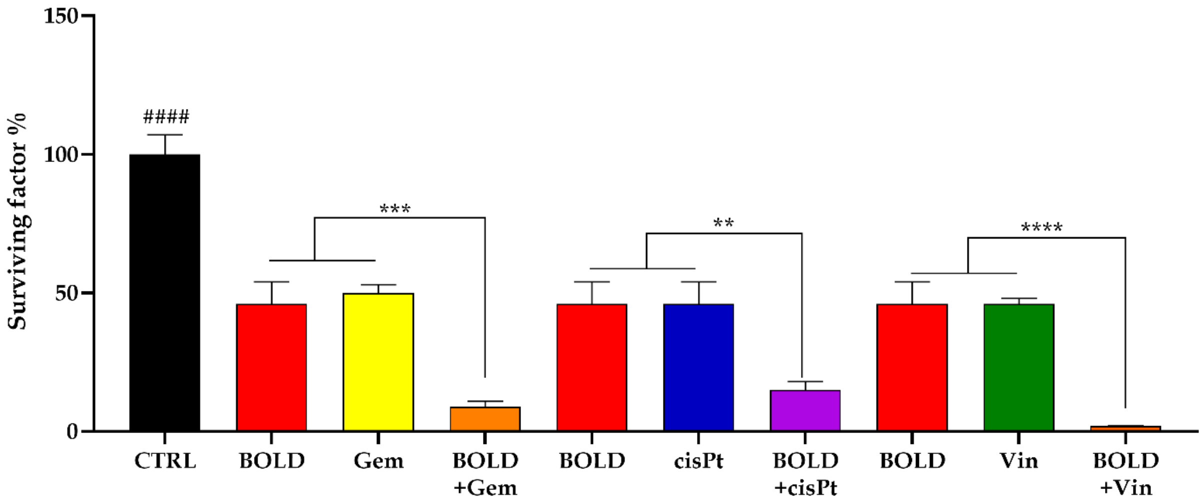

Suppl. 1 BOLD-100+drug combinations reduced colony formation in MSTO-211H cells. Colony formation ability measured in cells exposed for 48 h to BOLD-100, single drugs or the combinations BOLD-100+single drug. Data are means ± SD from 5 independent treatments, and indicated as surviving factor %. Symbols on bars indicate statistical differences: with respect to the CTRL (#### $p < 0.0001$, One-way ANOVA follow by Dunnett post-test); between combinations and their single components (** $p<0.01$, *** $p<0.001$, **** $p<0.0001$, One-way ANOVA follow by Dunnett post-test)

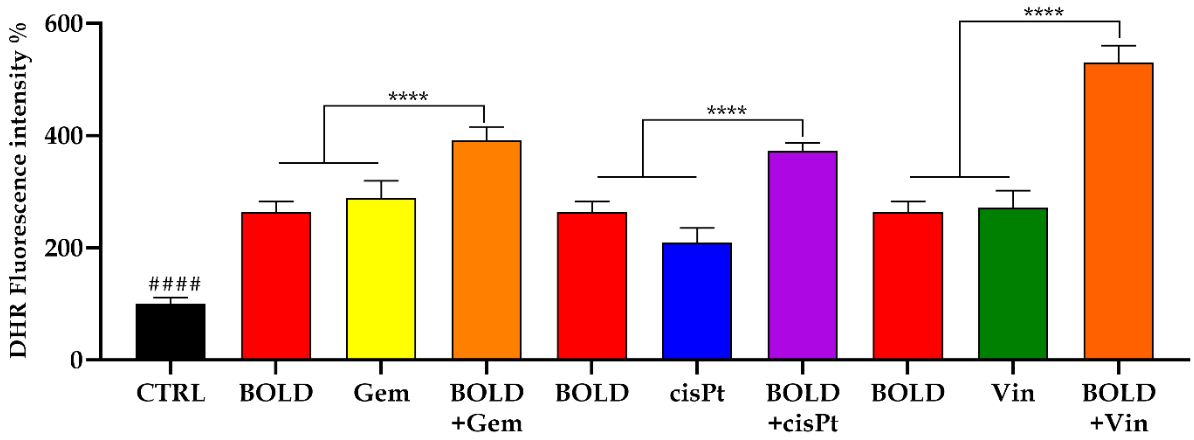

Suppl. 2 ROS alteration after BOLD-100+drug combinations treatment in MSTO-211H cells. ROS production evaluated as DHR fluorescence intensity recorded at 4 h in control cells (CTRL), or in cells incubated with BOLD-100, single drugs or the combinations BOLD-100+single drug. Data are means ± SD of DHR-123 fluorescence expressed as DHR fluorescence intensity %; n = 16 microplate wells from two different experiments. Symbols on bars indicate statistical differences: with respect to the CTRL (#### $p < 0.0001$, One-way ANOVA follow by Dunnett post-test); between combinations and their single components (**** $p<0.0001$, One-way ANOVA follow by Dunnett post-test).

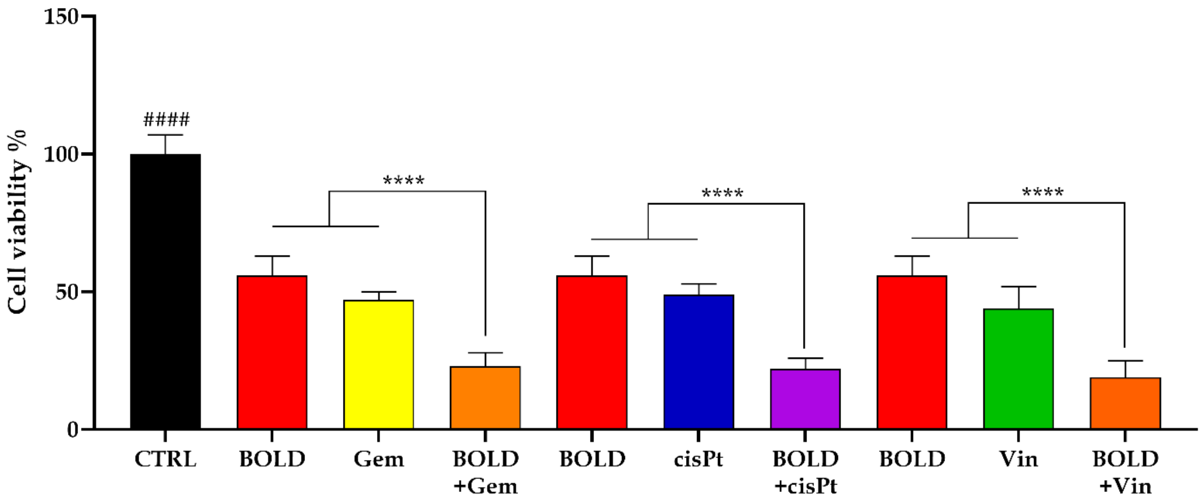

Suppl. 3 BOLD-100+drug combinations effect on cell viability evaluated at 72 h in MSTO-211H cells. Each value comes from the results of three independent experiments and is expressed as cell viability %. Symbols on bars indicate statistical differences: with respect to the CTRL (#### p < 0.0001, One-way ANOVA follow by Dunnett post-test); between combinations and their single components (**** p<0.0001, One-way ANOVA follow by Dunnett post-test).

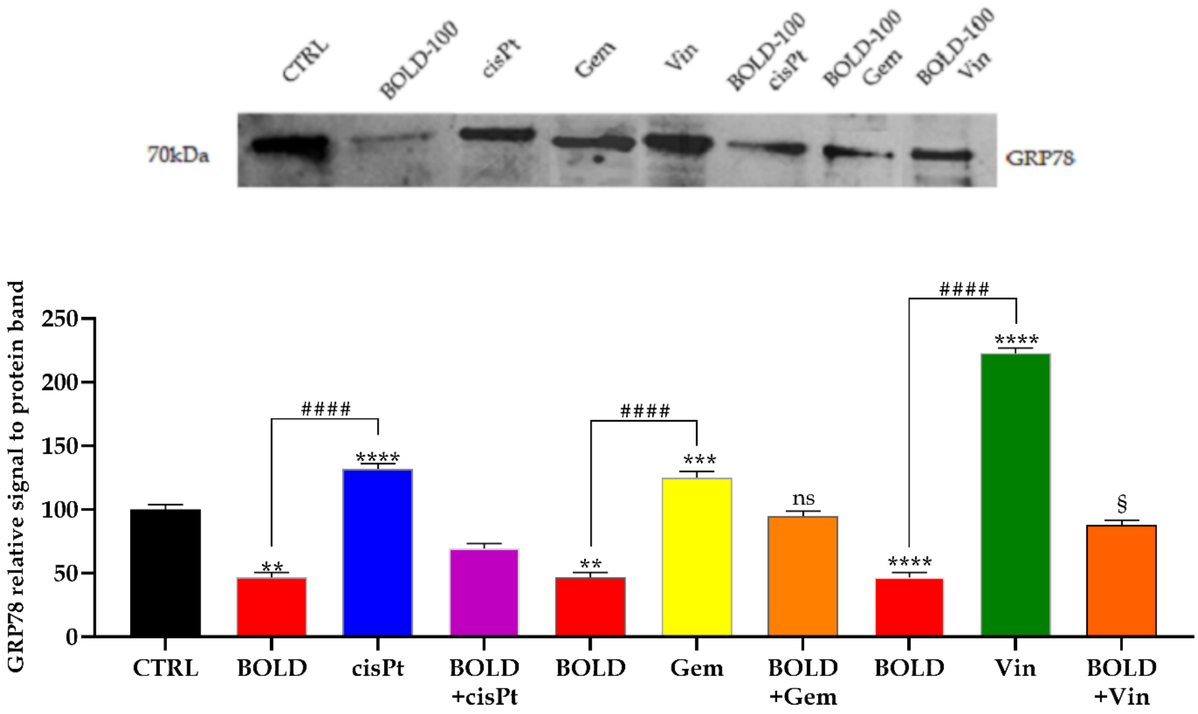

Suppl. 4 GRP78 protein expression in MSTO-211H cells after BOLD-100+drug combinations treatment. Blots in the upper part of the figure are representative of three; each lane was loaded with 20 µg of proteins, probed with anti-GRP78 mouse mouse-clonal antibody and managed as described in Materials and Methods. Symbols on bars indicate statistical differences: with respect to the CTRL (ns= not significant, § $p < 0.01$, if not indicated $p<0.0001$, One-way ANOVA follow by Dunnett post-test); between BOLD-100 and single drugs (#### $p < 0.0001$, One-way ANOVA follow by Dunnett post-test); between combinations and their single components (** $p<0.01$, *** $p<0.001$, **** $p<0.0001$, One-way ANOVA follow by Dunnett post-test).
